# Predictors of Critical Care Needs after IV Thrombolysis for Acute Ischemic Stroke

**DOI:** 10.1371/journal.pone.0088652

**Published:** 2014-02-12

**Authors:** Roland Faigle, Anjail Sharrief, Elisabeth B. Marsh, Rafael H. Llinas, Victor C. Urrutia

**Affiliations:** 1 Department of Neurology, Johns Hopkins University School of Medicine, Baltimore, Maryland, United States of America; 2 Department of Neurology, University of Texas Health Science Center, Houston, Texas, United States of America; University of Tübingen, Germany

## Abstract

**Background and Purpose:**

Intravenous (IV) tissue plasminogen activator (tPA) is the only Food and Drug Administration (FDA)-approved treatment for acute ischemic stroke. Post tPA patients are typically monitored in an intensive care unit (ICU) for at least 24 hours. However, rigorous evidence to support this practice is lacking. This study evaluates factors that predict ICU needs after IV thrombolysis.

**Methods:**

A retrospective chart review was performed for 153 patients who received intravenous tPA for acute ischemic stroke. Data on stroke risk factors, physiologic parameters on presentation, and stroke severity were collected. The timing and nature of an intensive care intervention, if needed, was recorded. Using multivariable logistic regression, we determined factors associated with requiring ICU care.

**Results:**

African American race (Odds Ratio [OR] 8.05, 95% Confidence Interval [CI] 2.65–24.48), systolic blood pressure, and National Institutes of Health Stroke Scale (NIHSS) (OR 1.20 per point increase, 95% CI 1.09–1.31) were predictors of utilization of ICU resources. Patients with an NIHSS≥10 had a 7.7 times higher risk of requiring ICU resources compared to patients who presented with an NIHSS<10 (p<0.001). Most patients with ICU needs developed them prior to the end of tPA infusion (81.0%, 95% CI 68.8–93.1). Only 7% of patients without ICU needs by the end of the tPA infusion went on to require ICU care later on. These patients were more likely to have diabetes mellitus and had significantly higher NIHSS compared to patients without further ICU needs (mean NIHSS 17.3, 95% CI 11.5–22.9 vs. 9.2, 95% CI 7.7–9.6).

**Conclusion:**

Race, NIHSS, and systolic blood pressure predict ICU needs following tPA for acute ischemic stroke. We propose that patients without ICU needs by the end of the tPA infusion might be safely monitored in a non-ICU setting if NIHSS at presentation is low.

## Introduction

Ischemic stroke is the second leading cause of death for people older than 60 years of age, with an estimated 795,000 new strokes each year in the United States alone [1], [2]. Thus, ischemic stroke is a costly health problem and a leading cause of long-term disability [3], [4]. Intravenous (IV) thrombolysis with recombinant tissue plasminogen activator (tPA) is the only Food and Drug Administration (FDA)-approved treatment for acute stroke and is currently the cornerstone of acute therapy for patients presenting within 4.5 hours of symptom onset [5]. In the United States, IV tPA for out-of hospital stroke patients is typically administered in the Emergency Department (ED) setting. Following IV tPA administration, it is currently recommended that all patients be observed closely for at least 24 hours with frequent vital sign checks and neurological examinations to allow for detection and early intervention of potential complications, such as symptomatic intracranial hemorrhage [6]. Post tPA care typically occurs in the setting of an intensive care unit (ICU) or a comparable stroke unit with ICU-like capabilities. While a subset of post IV tPA patients may require close monitoring in an ICU setting, it is unclear whether routine ICU-admission for all post tPA patients is medically necessary. Many patients are transferred out of the ICU after 24 hours without requiring any critical care intervention. This suggests that the unit is being used for “intensive monitoring” rather than “intensive care”. ICU resources are scarce and costly. Appropriate utilization of resources in the ICU is of vital importance to provide safe and cost-effective health care.

The purpose of the present study is to assess the need for routine ICU care in patients receiving IV tPA in the ED, and to identify variables that predict subpopulations of patients who may not require ICU care after IV tPA.

To our knowledge, this is the first study to explore the clinical predictors for ICU care in stroke patients post IV thrombolysis.

## Methods

### IV thrombolysis protocol

The Johns Hopkins Hospital and Johns Hopkins Bayview Medical Center are stroke centers certified by The Joint Commission. IV tPA is administered according to the American Heart Association (AHA) guidelines [6]. Post tPA monitoring with vital signs and neurological examinations conforms to the recommendations of the Brain Attack Coalition, which have become the standard of care for most stroke centers. All patients treated with IV tPA are monitored in the neurointensive care unit for at least 24 hours after initiation of thrombolysis, and all patients undergo imaging with either head CT or brain MRI 24 hours after treatment before being considered for transfer to the floor.

### Patients and Study design

This study was approved by the Johns Hopkins University School of Medicine Institutional Review Board. Data was obtained from prospectively collected de-identified databases of patients treated for stroke at The Johns Hopkins Hospital and Johns Hopkins Bayview Medical Center. A waver of consent was granted based on the following criteria based on 45 CFR 46.116: 1. The research involves no more than minimal risk to subjects; 2. The waiver will not adversely affect the rights and welfare of the subjects; 3. The research could not be practicably carried out without the waiver; and 4. The IRB will advise if it is appropriate for participants to be provided with additional pertinent information after participation. An IRB waiver of HIPAA privacy authorization was also granted to allow review of medical records to abstract data to de-identify for use in research.

We retrospectively analyzed the medical records of all patients who were treated with IV tPA within 4.5 hours of symptom onset in the ED, at Johns Hopkins Hospital or Bayview Medical Center, between January 2010 and March 2013. Patients with in-hospital strokes and patients who were subsequently transferred to or from other hospitals after tPA administration were excluded. Demographic data including age, sex, and race were collected for all patients. The presence of stroke risk factors including hypertension, hyperlipidemia, diabetes mellitus, smoking status, history of atrial fibrillation, and prior history of stroke, as well as the pre-hospital use of antiplatelet agents, anticoagulation, and statins were also recorded. National Institutes of Health Stroke Scale (NIHSS) is a standardized and easy to obtain tool used by providers and researchers in order to quantify stroke severity [7], [8]. Possible values on the NIHSS range from 0 to 42, higher values indicating increased stroke severity. NIHSS and the following physiologic parameters at presentation were recorded: blood pressure, international normalized ratio (INR), and estimated glomerular filtration rate (eGFR) by Modification of Diet in Renal Disease (MDRD) equation. The most likely stroke localization (anterior vs. posterior circulation) was recorded based on the patient's presenting symptoms.

The primary outcome was the need for a critical care intervention at any time point from the end of tPA infusion until transfer from the ICU to the floor. A critical care intervention was considered any therapy or intervention that required ICU resources, as defined previously [9], [10]. Specifically, ICU admission criteria included: uncontrolled hypertension requiring titration of IV antihypertensives, use of vasopressors either for symptomatic systemic hypotension or blood pressure augmentation, need for invasive hemodynamic monitoring, uncontrolled hyperglycemia requiring IV Insulin, respiratory compromise resulting in either initiation of bilevel positive airway pressure (BiPAP) or mechanical ventilation, arterial bleeding, management of cerebral edema and increased ICP, neurosurgical intervention such as decompressive craniectomy, or interventional angiography with or without intervention. Our definition of an ICU intervention also included patients with any event or complication that would require monitoring in an ICU setting even if no immediate ICU intervention was performed, such as progressive decrease in mental status with impaired airway protection, increasing oxygen requirement, or detection of potentially life-threatening arrhythmia. Patients who required ICU resources by the end of their tPA infusion or at any time over the next 24 hours were compared with those patients who did not have an ICU intervention during the same time period.

### Statistical Analysis

Statistical analysis was performed using STATA version 12 (*Stata Statistical Software: Release 12*. College Station, TX). A p-value of <0.05 was considered statistically significant. 95% confidence intervals are reported.

A multivariable statistical model of predictors of need for ICU resources was developed using statistically significant variables from the simple logistic regression analyses as well as other potentially clinically relevant variables such as age, time to tPA administration, and arterial circulation. A spline term was introduced for systolic blood pressure with cutoff of 140 mm Hg after descriptive analysis revealed a differential relationship between the primary outcome and systolic blood pressures below and above this number. Akaike information criterion (AIC) was used to aid in model selection.

Next, we estimated the risk of developing a need for ICU resources based on NIHSS greater than 10 at presentation using multiple logistic regression to control for other variables included in the selected model. This cutoff was determined based on descriptive analysis including graphical depiction of the data.

Finally, for the subset of patients who was without ICU need by the end of tPA infusion, we used Student's t-tests and Pearson's chi2 analyses to explore relationships between demographic variables and newly acquired need for ICU resources over the next 24 hours. We compared demographic variables and risk factor prevalence by need for ICU resources.

## Results

### Patient characteristics

A total of 154 patients received IV tPA in the ED of The Johns Hopkins Hospital and Johns Hopkins Bayview Medical Center for out-of hospital strokes between January 2010 and March 2013. One patient was excluded because he was transferred to another institution shortly after tPA administration, leaving 153 patients for further analysis. The average age was 65.4 years (range 28–94 years); 50.3% were female; and 47.1% were African American. One hundred twenty-one patients (79.1%) had a history of hypertension, 81 (52.9%) had a history of hyperlipidemia, 42 (27.5%) had a prior history of diabetes mellitus. The mean NIHSS was 9.8. In 129 patients (84.3%) the stroke was in the anterior circulation. The mean systolic blood pressure (SBP) at presentation was 162 mm Hg. Further baseline patient characteristics are presented in Table 1.

**Table 1:** Baseline characteristics by ICU need.

Characteristics	All patients *(n = 153)*	With ICU intervention *(n = 42)*	No ICU intervention *(n = 111)*	p-value
**Age** – years: mean (SD)	65.4 (16.4)	67.9 (15.2)	64.4 (16.8)	0.237
range	28–94	38–94	28–93	
**Race** – n (%)				**0.001**
African American	72 (47.1)	31 (73.8)	41 (36.9)	
Caucasian	79 (51.6)	11 (26.2)	68 (61.3)	
Other	2 (1.4)	0	2 (1.8)	
**Gender** – female n (%)	77 (50.3)	21 (50.0)	56 (50.5)	0.960
**NIHSS** – mean (SD)	9.8 (5.9)	12.8 (5.1)	8.6 (6.8)	**<0.001**
**BP** – mm Hg: mean (SD)				
SBP	162 (32.9)	171 (41.5)	159 (28.5)	0.078
DBP	91 (17.7)	95 (21.1)	89 (16.1)	0.138
**tPA time window** – n (%)				
0–3 hours	119 (78.8)	31 (73.8)	88 (79.3)	
3–4.5 hours	34 (22.2)	11 (26.6)	23 (20.7)	0.468
**Glucose** – mg/dl: mean (SD)	141 (66.1)	157 (80.9)	136 (58.8)	0.125
**eGFR <60 ml/min** – n (%)	55 (35.9)	16 (38.1)	39 (35.1)	0.734
**INR** – mean (SD)	1.07 (0.14)	1.1 (0.14)	1.05 (0.14)	0.098
**Distribution** – n (%)				0.784
Anterior circulation	129 (84.3)	35 (83.3)	94 (84.7)	
Posterior circulation	23 (15.0)	7 (16.6)	16 (14.4)	
Both circulations	1 (0.7)	—	1 (0.9)	
**Risk factors for stroke** – n (%)				
Hypertension	121 (79.1)	35 (83.3)	86 (77.5)	0.427
Hyperlipidemia	81 (52.9)	21 (50.0)	60 (54.1)	0.654
Diabetes mellitus	42 (27.5)	18 (42.9)	24 (21.6)	**0.009**
Atrial fibrillation	34 (22.2)	11 (26.2)	23 (20.7)	0.468
Prior ischemic stroke	37 (24.2)	11 (26.2)	26 (23.4)	0.721
Current smoking	43 (28.1)	5 (11.9)	38 (34.2)	**0.006**
**Medications** – n (%)				
Antiplatelet agent	70 (45.8)	18 (42.9)	52 (46.9)	0.658
Anticoagulation	15 (9.8)	5 (11.9)	10 (9.0)	0.591
Statin	65 (42.5)	15 (35.7)	50 (45.1)	0.297
**Total LOS** – days: mean (SD)	7.2 (7.15)	11.6 (9.48)	5.5 (5.19)	**<0.001**
**Mortality** – n (%)	8 (5.23)	8 (19.1)	—	**<0.001**

Forty-two patients (27.5%) required an ICU intervention either by the end of the tPA infusion or within the following 24 hours, while 111 patients (72.5%) did not have an ICU intervention during that time. The characteristics of these 2 groups including age, gender, race, NIHSS, stroke risk factors, medications for secondary stroke prevention, as well as blood pressure, blood glucose, INR, and GFR at presentation, are summarized in Table 1. The two groups were fairly similar, however, patients requiring an ICU intervention were more likely to be African American (73.8% vs. 36.9%), and have a higher NIHSS (mean 12.8 vs. 8.6). Patients requiring an ICU intervention had a longer total length of stay (mean days 11.6 vs. 5.5), and higher mortality rates (mean 19.1% vs. 0%), as compared to patients without ICU intervention.

### Variables predicting ICU care - Multivariable Analysis

**Figure 1:**
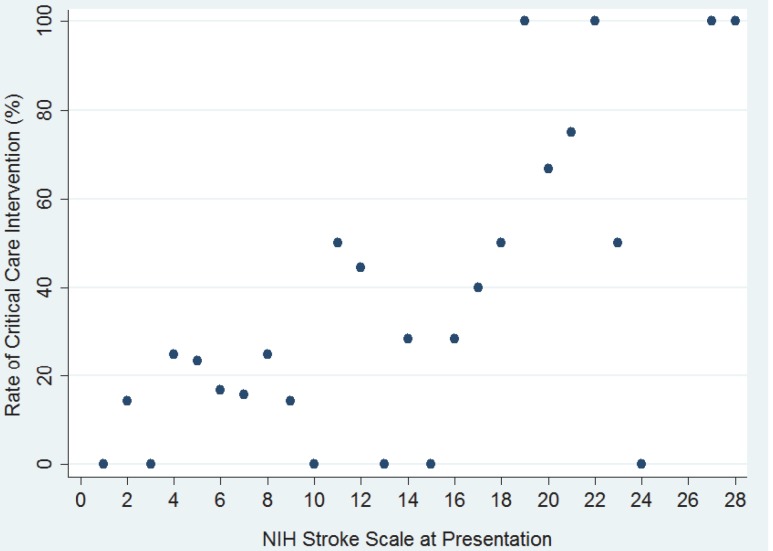
ICU needs by NIHSS after IV tPA. A scatter plot is shown illustrating the percentage of patients requiring ICU care by NIHSS at presentation.

In simple logistic regression analyses, African American race (OR 4.67 compared to Caucasian, 95% CI 2.12–10.30), history of diabetes mellitus (OR 2.72, 95% CI 1.27–5.81), systolic blood pressure (SBP) at presentation (OR 1.01, 95% CI 1.00–1.02), and NIHSS at presentation (OR 1.13 per 1 point increase in NIHSS, 95% CI 1.05–1.19) were all associated with requiring ICU level of care (Table 2). The probability of requiring ICU level of care in relationship to NIHSS at presentation is illustrated in Figure 1. The probability of requiring an ICU intervention was significantly higher for NIHSS≥10 compared to NIHSS<10 (40.6%, 95% CI 28.4–52.8 vs. 17.9%, 95% CI 9.9–26.1). Of note, current smoking was inversely related to requiring an ICU intervention (OR 0.26, 95% CI 0.09–0.71). There was an inverse linear relationship between systolic blood pressure and utilization of ICU resources for SBP<140 mmHg; however, for SBP>140 mmHg the probability of requiring ICU care increased with higher SBPs. Patient age, stroke localization, glucose levels at presentation, INR at presentation, and other evaluated stroke risk factors were not found to be predictors of requiring ICU care (Table 2).

**Table 2:** Logistic regression analyses: predictors of need for ICU intervention.

	Simple logistic regression			Multiple logistic regression		
Variable	OR	95% CI	p-value	OR	95% CI	p-value
**Age** (years)	1.01	0.99–1.04	0.233	0.99	0.95–1.03	0.547
**Race**						
Other	ref			ref		
African American	4.67	2.12–10.30	**<0.001**	8.05	2.65–24.48	**<0.001**
**Gender**						
Female	ref			ref		
Male	1.02	0.50–2.07	0.960	1.62	0.61–4.34	0.335
**NIHSS**	1.13	1.05–1.19	**<0.001**	1.20	1.09–1.31	**<0.001**
**Blood pressure**						
SBP	1.01	1.00–1.02	**0.039**			
≤140 mm Hg	0.95	0.90–1.00	0.051	0.93	0.89–0.98	**0.008**
>140 mm Hg	1.03	1.01–1.05	**0.002**	1.04	1.01–1.06	**0.001**
DBP	1.02	0.99–1.04	0.093			
**t-PA time window**						
0–3 hours	ref			ref		
>3 hours	1.36	0.59–3.10	0.469	2.25	0.67–7.60	0.191
**Glucose >140 mg/dl**	1.66	0.78–3.51	0.185			
**eGFR <60 ml/min**	1.14	0.54–2.37	0.734			
**INR>1.3**	2.48	0.78–7.85	0.124			
**Distribution**						
Anterior circulation	ref			ref		
Posterior circulation	1.18	0.45–3.10	0.744	3.53	0.84–14.7	0.084
**Risk factors for stroke**						
Hypertension	1.45	0.58–3.67	0.428	1.31	0.36–4.75	0.683
Hyperlipidemia	0.85	0.41–1.73	0.654			
Diabetes mellitus	2.72	1.27–5.81	**0.010**	2.82	0.99–8.00	0.052
Atrial fibrillation	1.36	0.59–3.10	0.469	1.09	0.31–3.86	0.893
Prior ischemic stroke	1.16	0.51–2.62	0.721			
Current smoking	0.26	0.09–0.71	**0.009**	0.22	0.06–0.83	**0.026**
**Medications**						
Antiplatelet agent	0.85	0.41–1.74	0.659			
Anticoagulation	1.36	0.44–4.26	0.592			
Statin	0.68	0.33–1.41	0.299			

In the multivariable model, African American race (OR 8.05, 95% CI 2.65–24.48) and NIHSS at presentation (OR 1.20 per point increase in NIHSS, 95% CI 1.09–1.31) remained statistically significant predictors of requiring ICU level of care by the end of the tPA infusion or in the ensuing 24 hours (Table 2). The relationship between utilization of ICU resources for SBP less than 140 mmHg (OR 0.93 per point increase in SBP<140 mmHg, 95% CI 0.89–0.98) and greater than 140 mmHg (OR 1.04 per point increase in SBP, 95% CI 1.01–1.06) also remained significant in the multivariable analysis. Diabetes mellitus showed a trend toward significance (OR 2.82, 95% CI 0.99–8.0). Smoking at presentation remained inversely related to requiring ICU care (OR 0.22, 95% CI 0.06–0.83).

When NIHSS was dichotomized (≥10 versus <10 based on the observation that those with NIHSS≥10 were more likely to require an ICU intervention; (Figure 1)), patients with an NIHSS≥10 had a 7.7 (95% CI 2.56–22.92) times higher risk of requiring ICU resources either by the time of tPA completion or the ensuing 24 hours compared to patients who presented with an NIHSS of less than 10 (p<0.001) after controlling for the variables included in the multivariable model. For NIHSS≥10 the OR for developing an ICU indication increased by 1.21 for every point increase in NIHSS (p = 0.018), while there was no significant increase per point NIHSS for patients presenting with NIHSS of <10 (p = 0.100).

### Identifying a critical time window of requiring ICU care

**Figure 2:**
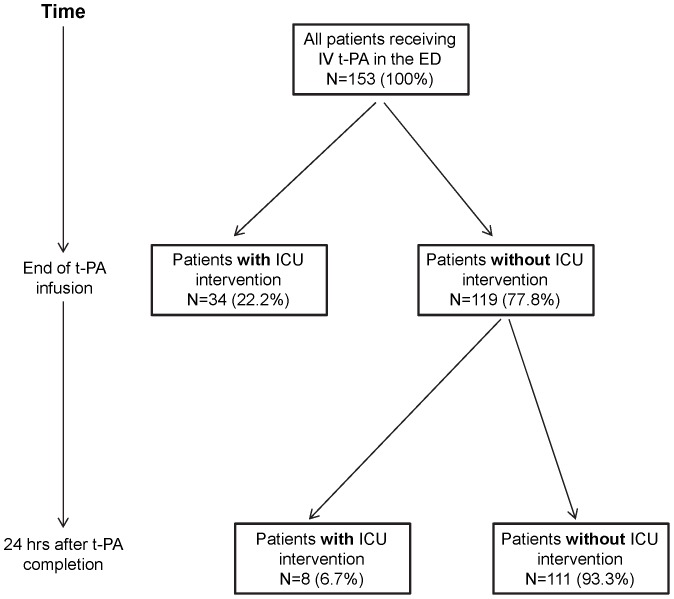
Timing of ICU needs after IV tPA. A flowchart is shown illustrating the need for critical care interventions over time in all patients receiving IV tPA.

To identify a potentially critical time window during which most patients with critical care needs acquire their ICU indication, we divided the population of 42 patients with an ICU intervention into 2 populations depending on the time point by which they required ICU level of care. Thirty-four of 42 patients (81.0%, 95% CI 68.8–93.1) requiring ICU resources had critical care needs by the end of tPA infusion, suggesting that most patients who undergo an ICU intervention will declare their needs early on. The most common ICU indications by the end of the tPA infusion were: IV drips for uncontrolled hypertension (13/34), management of respiratory compromise (12/34), and intra-arterial therapy (6/34). Of the 119 patients without ICU intervention by the end of tPA infusion, 111 (93.3%, CI 88.7–97.8) patients did not require any further ICU level of care, while 8 (6.7%, CI 2.2–11.3) patients required an ICU intervention at any point over the next 24 hours (Figure 2). The ICU interventions for patients who required critical care needs within the 24 hours after completion of tPA infusion included management of cerebral edema (4/8), use of vasopressors for blood pressure augmentation (2/8), and management of symptomatic intracranial hemorrhage (2/8).

### NIHSS and Diabetes Mellitus are associated with critical care needs in patients without ICU indication by the end of tPA infusion

We compared the characteristics of the 8 patients with new ICU requirements between the end of tPA infusion and the following 24 hours with the 111 patients who remained without need for ICU resources (Table 3). The two groups were fairly similar, however, the mean NIHSS of patients requiring ICU level of care was 17.3 (95% CI 11.5–22.9), compared to 8.6 (95% CI 7.6–9.6) in patients with no ICU requirements (p<0.001). Only 1 of the patients with new ICU requirements had an NIHSS<10 (8). This patient had a cerebellar stroke and required decompressive suboccipital craniectomy for cerebral edema. Patients with newly acquired ICU needs were more likely to have diabetes mellitus (p = 0.009), while there was no significant difference in the presence of other common stroke risk factors including hypertension (p = 0.131), hyperlipidemia (p = 0.25), atrial fibrillation (p = 0.774), smoking (p = 0.206), or prior stroke (p = 0.095) (Table 3).

**Table 3:** Demographic and clinical variables of patients with newly acquired ICU needs after completion of tPA infusion.

Characteristics	All patients *(n = 119)*	New ICU intervention by 24 hours *(n = 8)*	No ICU intervention *(n = 111)*	p-value
**Age** – years: mean (SD)	64.7 (16.9)	68.8 (18.9)	64.4 (16.8)	0.487
range	28–94	38–94	28–93	
**Race** – n (%)				0.348
African American	71 (59.7)	3 (37.5)	68 (61.3)	
Caucasian	46 (38.7)	5 (62.5)	41 (36.9)	
Other	2 (1.6)	0	2 (1.8)	
**Gender** – female n (%)	59 (49.6)	3 (37.5)	56 (50.5)	0.479
**NIHSS** – mean (SD)	9.2 (5.67)	17.3 (6.81)	8.6 (5.14)	**<0.001**
**BP** – mm Hg: mean (SD)				
SBP	159 (28.5)	159 (30.2)	159 (28.5)	0.995
DBP	90 (16.2)	98 (15.5)	89 (16.1)	0.122
**tPA time window** – n (%)				
0–3 hour	95 (79.9)	7 (87.5)	88 (79.3)	
3–4.5 hours	24 (20.1)	1 (12.5)	23 (20.7)	0.576
**Glucose** – mg/dl: mean (SD)	138 (59.6)	172 (63.7)	136 (58.8)	0.093
**eGFR <60 ml/min** – n (%)	41 (34.5)	2 (25.0)	39 (35.1)	0.560
**INR** – mean (SD)	1.06 (0.14)	1.06 (0.05)	1.05 (0.14)	0.745
**Distribution** – n (%)				0.950
Anterior circulation	101 (84.9)	7 (87.5)	94 (84.7)	
Posterior circulation	17 (14.3)	1 (12.5)	16 (14.4)	
Both circulations	1 (0.8)	0	1 (0.9)	
**Risk factors for stroke** – n (%)				
Hypertension	94 (79.0)	8 (100)	86 (77.5)	0.131
Hyperlipidemia	66 (55.5)	6 (75.0)	60 (54.1)	0.250
Diabetes mellitus	29 (24.4)	5 (62.5)	24 (21.6)	**0.009**
Atrial fibrillation	25 (21.0)	2 (25.0)	23 (20.7)	0.774
Prior ischemic stroke	30 (25.2)	4 (50.0)	26 (23.4)	0.095
Current smoking	39 (32.8)	1 (12.5)	38 (34.2)	0.206
**Medications** – n (%)				
Antiplatelet agent	58 (48.7)	6 (75.0)	52 (46.9)	0.124
Anticoagulation	10 (8.4)	0	10 (9.0)	0.375
Statin	54 (45.4)	4 (50)	50 (45.1)	0.786

## Discussion

In the United States, IV thrombolysis for patients with acute ischemic stroke follows standardized protocols. All post IV tPA patients are triaged to an ICU or stroke unit with intense monitoring capabilities for at least 24 hours, regardless of patient demographics, ED course, or other variables at presentation. However, little data exist as to whether ICU level of care is medically necessary or a cost-effective use of ICU resources.

The present study identifies NIHSS, African American race, and SBP at presentation as independent predictors of ICU resource utilization by the end of the tPA infusion and/or over the following 24 hours. It also suggests that history of diabetes mellitus is a possible predictor, although this did not reach statistical significance in multivariable analysis (p = 0.052). Our data show that the end of the tPA infusion may serve as a critical triage time point for patients after IV thrombolysis. We propose that patients who do not require ICU resources by the end of tPA infusion may safely be monitored in a non-ICU setting if their presenting NIHSS is <10.

In stroke patients, the NIHSS correlates with infarct size and clinical severity [7], [11]. Thus, our finding of NIHSS predicting ICU resource utilization might be related to an increased risk of complications associated with increased infarct size, such as cerebral edema, symptomatic hemorrhagic transformation, or airway compromise.

Interestingly, race remained a significant predictor of ICU resource utilization after controlling for NIHSS and systolic blood pressure at presentation in the multivariate model. Several previous studies have demonstrated racial disparities with regards to stroke incidence, underlying stroke mechanism, and access to health care resources. African Americans have an overall increased stroke incidence compared to Caucasians [12], [13], and have a lower prevalence of atrial fibrillation [14], [15], and extracranial carotid disease [16], [17]. Higher NIHSS is more often seen in the context of large vessel and cardioembolic strokes while small vessel strokes tend to present with lower NIHSS [18], [19]. Although the underlying stroke mechanism was not formally collected in this study, there was no difference in NIHSS by race in our population (mean NIHSS in African Americans 9.4, 95% CI 8.1–10.7 vs. 10.2 in non-African Americans, 95% CI 8.8–11.5). Therefore, the underlying stroke etiology is unlikely to explain our observed racial disparity. Resistant hypertension is more commonly seen in African Americans [20]. Since a significant proportion of ICU interventions were aimed at acute blood pressure control, patients with acute refractory hypertension at the time of presentation would be more likely to require ICU resources for blood pressure control. However, it is unclear how acutely elevated blood pressure relates to chronic outpatient blood pressure control, and whether race might contribute to acutely refractory hypertension in the setting of an ischemic stroke. Systolic blood pressure was recently shown to differentially impact the risk for stroke in African Americans as compared to Caucasians [21]. Stroke incidence was significantly higher in African Americans, even at comparable blood pressure values, suggesting increased susceptibility to elevated blood pressures [21]. Differential effects of blood pressure beyond “blood pressure at presentation” may account for the higher rate of complications and the increased need for critical care interventions in African Americans after IV thrombolysis observed in our study. In addition, the presence of other factors and co-morbidities not controlled for in the present study, such as long-term diabetes or lipid control prior to hospitalization, might explain the increased likelihood of African Americans requiring ICU resources.

Smoking is a well established independent risk factor for ischemic stroke [22], [23]. In our study, smoking was associated with a decreased risk of requiring ICU resources after IV tPA administration, even after controlling for other variables. While this finding was somewhat unexpected, it is not inconsistent with previous reports. Ovbiagele and Saver showed that smokers with acute ischemic stroke who received IV thrombolysis had greater improvement in their NIHSS from baseline and better early outcomes compared to non-smokers [24]. The authors hypothesized that the underlying pathophysiology of vascular occlusions in smokers may be thrombogenic rather than atherogenic, and the resulting thrombus may therefore be more susceptible to lysis by tPA as compared to platelet-rich clots found in non-smokers. Similarly, a more recent study showed that smokers had increased recanalization and reperfusion rates and better functional outcome at 3 months after IV tPA for acute ischemic stroke compared to non-smokers [25].

In our study, only 6.7% of patients without ICU needs by the end of the infusion went on to develop an ICU indication over the following 24 hours. These patients had higher NIHSS and were more likely to have a history of diabetes mellitus. This is consistent with previous studies demonstrating that strokes in patients with a history of diabetes are associated with higher mortality rates, higher rates of hemorrhagic transformation after IV tPA, and overall poorer outcomes as compared with nondiabetics [26], [27].

The development of a patient profile that enables clinicians to identify those patients who are most likely to require ICU resources after IV tPA administration would allow for optimization of resource utilization and increasing cost effectiveness. Similar approaches have been undertaken for other neurological and neurosurgical patient populations, including patients with traumatic brain injury [28], traumatic intracranial hemorrhage [29], post-brain biopsy [30], and post elective craniotomy [31]. Sparing a distinct patient population an ICU admission may result in decreased hospital costs and reduced length of hospital stay, mainly by reducing the number of transfers between different medical teams, enabling a more efficient evaluation by ancillary teams, such as physical and occupational therapy, and expediting the completion of the work-up. Several institutions currently monitor post-tPA patients in intermediate care units or dedicated stroke units. However, in order to comply with current guidelines, these patients are required to undergo intense monitoring often with one-to-one nursing care. Independent of physical location of the patient, the high frequency of neurological exams and vital sign checks dictates resource-intense monitoring similar to ICU care. Our findings potentially identify a group of post-tPA patients for which allocation of scarce ICU resources is appropriate.

Our study has several limitations. We excluded patients with in-house strokes, since those patients are likely to have multiple and complex medical problems. In addition, this is a retrospective analysis of a small number of patients over the course of 3 years. Additionally, indications for ICU admission and interventions may differ among institutions across the US, and the model described in this study might therefore not be valid in institutions where ICU admission criteria differ significantly from ours. Further prospective studies are needed for validation of our model, and to ensure that long-term outcomes in patients not receiving ICU care post tPA are similar to the current standard of care. Despite these limitations, the present data highlight predictors of ICU requirement after IV tPA for patients with acute ischemic out-of hospital stroke, and identify a subgroup of patients that may not need ICU care.
